# Technique of Making Urethral Injections

**Published:** 1894-12

**Authors:** 


TECHNIQUE OF MAKING URETHRAL INJECTIONS.—Guiard (*Annales des Maladies des Organes Genito-Urinaire*, June, 1894) gives the results of his investigations concerning the urethra and its medication. The capacity of the urethra had been stated by Jarnin and Leprevoct to be from five to eight grammes, therefore it was held that a urethral syringe should not hold more than five or six grammes, equal to about one and a half drachms. Later, it was shown that posterior urethritis, particularly late in the disease, was far more frequent than formerly supposed. The author by experimenting on the living subject found that the urethra would always hold eight to ten grammes (two to two and a half fluid drachms), and more often twelve to fifteen grammes, and sometimes sixteen to seventeen. As the patient could tell when the sphincter was forced, this was avoided. These deep injections are only called for when definite symptoms have already demonstrated that the posterior region of the urethra is already affected.

In order to administer these deep injections, the author uses a syringe of twenty grammes (five drachms) capacity. When it is desired to overcome the sphincter, gentle pressure is made when the liquid will enter. In an experience of ten years he has never had any accidents, and only encountered one case in which the sphincter would not relax. It is better to give the injections when the patient is lying down than when he is standing up. The requirements of an effective injection is that it shall reach all the diseased parts. To do this a syringe of twenty grammes (five fluid drachms) capacity should be used, and the injection of its entire contents, if carefully done, is easy, and causes no inconvenience.—*Ex.*

